# Cavitation erosion from single acoustically driven bubbles

**DOI:** 10.1016/j.ultsonch.2026.107740

**Published:** 2026-01-07

**Authors:** Jaka Mur, Vid Agrež, Claus-Dieter Ohl, Rok Petkovšek

**Affiliations:** a Faculty of Mechanical Engineering, University of Ljubljana, Aškerčeva 6, SI-1000 Ljubljana, Slovenia; b Faculty of Natural Sciences, Institute for Physics, Otto-von-Guericke-University Magdeburg, Universitätsplatz 2, 39106 Magdeburg, Germany

## Abstract

Acoustic cavitation is achieved by exciting mechanical vibrations at ultrasonic frequencies, which in turn cause the formation of bubble clouds, followed by flows and emulsification. Typically, the effects of acoustic cavitation clouds on cleaning and erosion are difficult to predict or model due to the complex interactions among numerous bubbles. Systematic studies of acoustic cavitation bubbles are simplified by using single cavitation bubbles as a means of controlled cavitation, owing to their precisely defined timing and properties, which can be induced within an acoustic field by seeding a small laser-induced bubble within it. This work presents findings on the erosion of solid surfaces initiated by a single acoustic bubble. Optical seeding of a small cavitation bubble is combined with acoustic driving under a sonotrode tip to generate a single, controlled, and isolated acoustically driven bubble oscillating near a solid boundary. The phase delay and spatial coordinates of optical seeding within the acoustic field are explored to achieve repeatable acoustic bubble behavior with multiple expansion–collapse cycles as a single bubble before transitioning into a bubble cloud composed of multiple smaller bubbles. Using an ultra-high-speed camera and a hydrophone pressure sensor, bubble collapses are quantified in terms of shockwave energy and position. Finally, the resulting erosion patterns on the aluminum surface are measured using confocal laser surface scanning after multiple event repetitions. This technique enables the study of erosion patterns produced by temporally and spatially confined acoustically driven bubbles.

## 1 Introduction

Ultrasound, the travelling pressure waves at frequencies beyond the hearing range, can cause bubble formation in liquids when the pressure oscillation amplitude is above the critical threshold. The formation of bubbles, followed by their growth and collapse, is called acoustic cavitation [1]. These bubbles typically form bubble clouds regions of intense pressure-wave driving, which are the basis for a wide variety of uses, typically liquid processing relying on the oscillating vibration of the sonotrode tip. At the tip, bubble clusters form, then violently collapse [2], making acoustic cavitation suitable, for example, for surface cleaning [3], emulsification [4], sonochemistry [5], and even therapeutic treatment [6], [7]. Precise control of acoustic cavitation is difficult to achieve as complex bubble–bubble interactions prevent predictable and fully repeatable behavior of the bubble cloud when using only the typically accessible control variables, namely the sonotrode driving amplitude and number of oscillation cycles. The mechanical inertia and resonance quality of the sonotrode causes delayed bubble cloud formation at the sonotrode tip at the start of driving and prolonged formation after the end of driving. The tip continues with slowly-damped oscillations even after the driving signal is stopped [1] for several tens of milliseconds. Therefore, the two control parameters are not sufficient to prepare similar bubble clouds for repeated runs, while the complex interactions within the acoustic bubble clouds also make them difficult to numerically simulate.

Due to the generally chaotic nature of acoustic cavitation bubble clouds, single bubbles in a periodic pressure field offer a clearer look into bubble dynamics. The emission of light from collapsing bubbles, i.e., sonoluminescence [8], [9], received the most attention in the early works in the field [10] due to the possible enhancement of liquid energy focusing during the bubble collapse. Nowadays, research motivation stems mainly from the use of microbubbles as ultrasound contrast agents [11], [12] and drug delivery mediators [13], [14], but also from the possibility of erosion through studies of bubbles near rigid boundaries [15], [16], [17].

A precise and repeatable way to inject a small single bubble into a periodic pressure field is laser-induced single cavitation bubble generation. Laser-induced cavitation bubbles have been used as a method of choice for applications in material processing [18] and medicine [19]. Their main advantages are the precisely defined timing and spatial confinement, which have led to their wide use in fundamental studies ranging from bubble dynamics and related phenomena [20], shock wave [21] and plasma formation [22], sonoluminescence studies [23], to bubble collapses in various geometries [24]. These collapses attracted attention due to the intensive liquid energy focusing in the region of collapse, leading to the aforementioned sonoluminescence in a free liquid environment and material erosion through collapses near a surface under correct conditions [25]. The versatility of the method is somewhat limited by the laser focusing conditions and the fact that typically only a single oscillation of the bubble is repeatably obtained. A single laser-induced bubble in an acoustic pressure field is a known phenomenon [8], [10], for which existing studies have established the effect of acoustic pressure on the time of collapse and bubble radius, as well as have employed different methods for seed bubble inception [26]. The investigations included the effect of varying the acoustic phase with respect to bubble generation.

This study builds on the foundations of previous studies of single bubble dynamics in an acoustic field [8], [9], [10], and partially follows the method described in a previous study of controlled bubble cluster formation in the gap below the sonotrode [27]. The cited work presents a method in which a laser‑induced single cavitation bubble, once introduced into an ultrasonic field, splits and develops into a cluster of bubbles that persists over numerous acoustic cycles. The previous work emphasizes the controlled formation and dynamics of bubble clusters under acoustic driving, rather than maintaining a single bubble over many cycles. The present study optimizes optical bubble seeding for tailored acoustic bubble behavior before its transition into a cloud of bubbles, with the goal of systematically relating it to material erosion. An acoustic field generated by a sonotrode is used at amplitudes below the threshold for acoustic cavitation [1], together with a bubble optically seeded at a specific position and phase in the gap between the sonotrode tip and the solid plate. The pulsed laser focus is radially centered below the sonotrode. The laser pulse energy is optimized for repeatable generation of relatively small cavitation bubbles compared to the sonotrode tip and gap sizes. Overall, the experiment is designed to generate a single, controlled, and isolated acoustically driven bubble oscillating near a solid boundary, before it transitions to a bubble cloud attached to the sonotrode tip. For the first time, material erosion originating from a single acoustically driven bubble repeatedly collapsing near a solid material surface is observed and quantified, as opposed to previous studies that observed average ensemble effects stemming from bubble clouds [28].

The investigation begins with the effects caused by the bubble seeding phase with respect to the acoustic pressure field on single acoustically driven bubble dynamics. The bubble dynamics are imaged with a high-speed camera and emitted collapse shock waves are measured with a hydrophone. Further, the gap size and the optical-seeding position within the gap are studied, with the goal of obtaining repeatable experimental conditions that maximize the potential for erosive effects. For this, several requirements are identified. First, the acoustically driven bubble should persist as a single bubble, i.e., a single connected gas volume, through multiple acoustic oscillations as opposed to forming a bubble cluster or even a bubble cloud. A bubble cluster or cloud spreads the available energy over a larger area. Second, the position of bubble collapses should be as close as possible to the solid surface below the sonotrode. Finally, the shock waves emitted at these collapses should be as strong as possible, indicating a highly constrained collapse volume. As a result, a repeatable and controlled regime is identified for driving a single bubble through multiple oscillations near the solid surface inside the narrow gap. Lastly, the bubble dynamics leading to erosion of polished aluminum plates are measured and quantified, as well as the resulting surface properties after repetitive nucleation of optical bubbles causing acoustic cavitation erosion.

## 2 Methods

The experimental setup consisted of an ultrasound source, an in-house built pulsed laser source for seeding bubbles, and the imaging setup able to record the events using high-speed imaging supported by an in-house built laser-based illumination system.

### 2.1 Acoustic cavitation methods

**Fig. 1:**
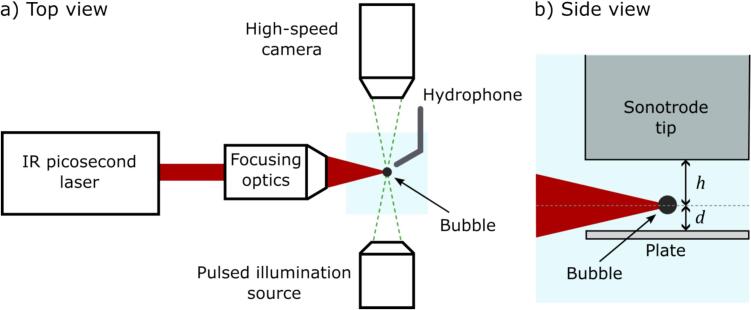
a) Schematic representation of the experimental setup in top view, establishing the general arrangement of measurement and cavitation equipment. the sonotrode tip is positioned centered above the bubble, and the solid plate below it, as shown in the side view representation of the experiment volume in panel b). The latter view corresponds to the camera perspective.

The ultrasonic emitter for acoustic cavitation was a sonotrode horn (Bandelin SONOPULS TS 102 using a MS 72 tip) connected to an electric amplifier. The 2 mm diameter sonotrode tip and the solid plate sample were inserted on top of each other into a 3D printed cuvette with a volume of 90 ml, while being vertically separated by 0.3 – 0.8 mm. The schematic of the crucial experiment parts is presented in Fig. 1a and a detailed view inside the gap presented in Fig. 1b. The sonotrode driving was matched to its resonance frequency of 20.06 ± 0.02 kHz using an amplified sinusoidal electric signal. A small drift in the resonance frequency is caused by variations of temperature of the water and air and was compensated for during the experiments. The voltage amplitude needed for driving the sonotrode in a regime of continuous acoustic cavitation was first experimentally defined. For all further measurements, the voltage amplitude was kept at around 80 % of the threshold amplitude. Therefore, in absence of optical bubble seeding no acoustic cavitation was initiated. At this driving, the vertical sonotrode tip movement with an amplitude of 25 ± 3  µm was measured using the microscopy imaging combined with the high-speed cameras.

Acoustic pressure at small distances below the sonotrode tip cannot be obtained experimentally, as a direct measurement is not possible due to the physical size constraints and pressure field disruption by the hydrophone tip. We obtained the acoustic pressure estimation through simulations based on the finite element solver in COMSOL Multiphysics together with the acoustics toolbox, similarly to our previous work [27]. The experimentally measured sonotrode tip oscillation amplitude was set as a boundary condition, assuming smooth sinusoidal oscillation that best fit the high-speed imaging measurements. The sonotrode tip is made of grade 5 titanium (TiAl6V4 − 3.7165) with an acoustic impedance of around 27 MPa·s/m, giving 90 % sound reflection on the water-titanium boundary. The simulation output is a pressure amplitude of 230 ± 15 kPa at the position 350 µm away from the sonotrode tip along the symmetry axis of the sonotrode for 25 µm sonotrode oscillation amplitude. The obtained values come close to reported thresholds for acoustic cavitation, see Ref. [29]. The absence of self-triggered acoustic cavitation was also confirmed experimentally numerous times during the experiment runs.

### 2.2 Optical cavitation and imaging methods

**Fig. 2:**
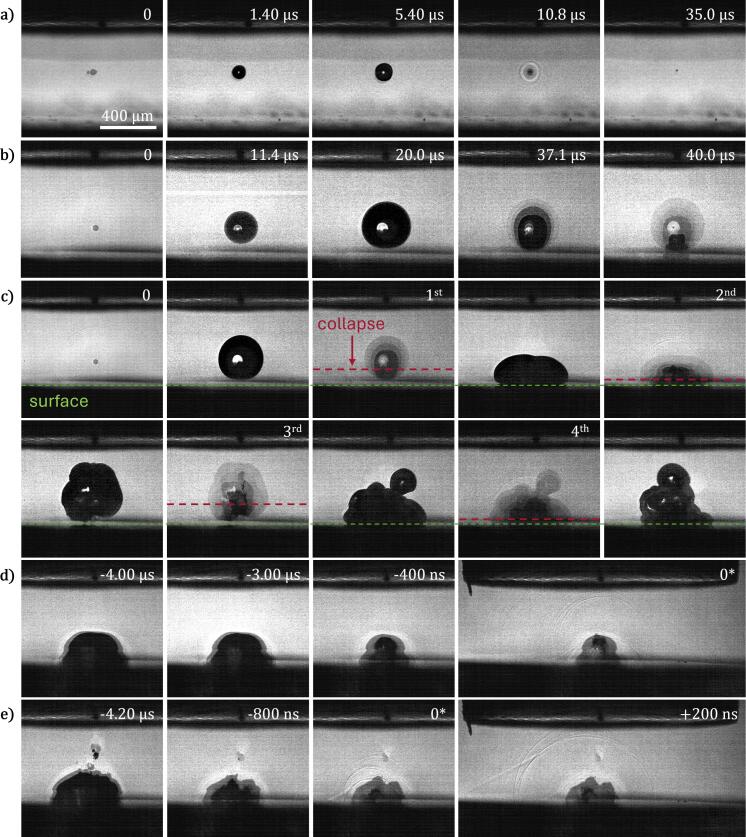
High-speed imaging of the bubble dynamics in the gap between the sonotrode (top) and a solid glass plate (bottom). a) The reference laser-induced cavitation bubble in absence of an external pressure field is shown. The bubble lifetime in this case is 10.8 µs. b) The first oscillation of a laser-induced cavitation bubble affected by an external pressure field is shown. The generation phase is close to optimum for a maximal bubble response. c) Selected frames showing the first four bubble oscillations from nucleation to the fourth rebound. Surface position is marked with a green dashed line, while the estimated collapse positions are marked with orange dashed lines. The second and fourth collapses happen close to the solid plate surface. This is detailed in d) and e) with frames taken from a 5.0 MFPS recording showing signature moments of the second and fourth collapse. Scale bar is valid for all panels. Time 0* is defined as the frame closest to the minimal volume bubble, while time 0 is the frame closest to laser-induced bubble seeding. Videos are available as Supplementary Material.

Optical cavitation is initiated by focusing a home-build picosecond fiber laser (wavelength 1030 nm, pulse duration of 60 ps) [30] in the gap between the sonotrode tip and the solid plate. Bubbles are formed at a laser energy in the range of 60–100 μJ, depending on exact focusing conditions, specifically partial beam clipping by the solid plate. The laser is focused in the cuvette through a long working distance microscope objective (20x magnification, NA = 0.45). The laser energy was tuned for each experiment setting to result in a laser-induced bubble with a maximum radius of 65 ± 3  µm, and a corresponding first oscillation period of around 10.5 ± 0.5 µs without operating the sonotrode, i.e., without the acoustic field (oscillation shown in Fig. 2a). The bubble is generated at a distance *h* from the sonotrode tip, and a distance *d* from the solid plate surface below the bubble (see Fig. 1b). The gap height is H=d+h. A sequence of the seeding bubble oscillation without ultrasound driving is shown in Fig. 2a where h≈300μm and d≈400μm. For a simplified and comparable presentation of the bubble position within the gap we use a relative bubble position definition $d_{\mathrm{rel}}=d/h$, which is in this case 1.33. The first bubble oscillation changes significantly with the ultrasound driving enabled (Fig. 2b), becoming orders of magnitude larger in volume compared to no acoustic driving.

The bubble dynamics is observed with two high-speed cameras interchangeably. The first high-speed camera (Photron Nova S9) can record many frames and thus for dynamics over a long duration, however, is limited to 120 kFPS due to resolution restrictions. The second high-speed camera (Specialized Imaging Kirana 7 M) is used to capture the bubble dynamics only for the first few oscillations, collapse positions, and shock wave emission. This camera can record up to 5 million frames per second (MFPS), yet only 180 frames in one recording. The two high-speed cameras were combined with microscopy objectives with 5x and 10x magnification, respectively, ensuring corresponding imaging resolution of 4.0 µm/pix (Nova S9 camera with 5x objective) and 3.0 µm/pix (Kirana 7 M camera with 10x objective). A home-built short-pulse illumination system was used for synchronized illumination of the high-speed camera frames. Each camera frame is illuminated with one light pulse about 3–4 ns in duration.

The timing of the laser with respect to the acoustic cycle is critical for repeatable bubble dynamics. First, the sonotrode oscillations were started and waited for around 2000 acoustic cycles for a stable vibration amplitude due to the high resonance quality of the sonotrode. After this time, the sonotrode oscillation is at the desired amplitude and surrounded by a fully developed acoustic field. Therefore, about 100 ms of sonotrode oscillations are waited prior to the optical initiation of the cavitation bubble. The slower high-speed camera was used to record this interval to make sure that no acoustic cavitation occurred, to establish the general experimental conditions, and validate the numerically obtained pressure estimates. To control the phase between the acoustic field and the optical bubble generation, we used a function generator for both the laser pulse emission timing and driving the sonotrode. The faster high-speed camera was used to capture the first 180  µs of the bubble dynamics when recording at 1 MFPS, in some cases for 360 µs when recording at 500 kFPS, capturing the dynamics for up to five full sonotrode oscillations.

The experiment was optimized for the generation and perpetuation of a single acoustically driven bubble that does not form a cavitation bubble cluster or cloud for at least 2–4 sonotrode oscillation periods. Due to the close solid surface proximity, the bubble shape is most of the time far from spherical. We define a single bubble as a single connected gas volume when at its maximum expansion, regardless of its shape. Any temporary bubble breakups following the bubble collapse are not considered as a bubble cluster, if those parts reconnect during the expansion phase. In the single bubble case, the energy focusing during the bubble collapse is by far the strongest, as the whole bubble volume collapses to a single spot, as opposed to multi-bubble collapses.

An example of the bubble shape evolution from inception up to the fourth re-expansion is presented in Fig. 2c. On the frames showing the collapse, it appears that many bubbles are overlaid at the same position. Unfortunately, this is an artifact of the camera, and only object with the darkest outline is the actual bubble. The most important observation from the image sequence is that the collapse positions alternate between close and far distance from the solid plate. Here the green and orange dashed lines represent the surface and collapse position, respectively. The first and the third collapse occur relatively far from the surface, i.e., 100–300 µm away, while the second and the fourth collapses close, i.e., less than 50 µm away. The fifth collapse is then again found further away from the surface. The second and fourth collapses are that of a large volume bubble close to the surface, presumably creating nearly ideal conditions for surface erosion to occur. A high-speed video sequence of second and fourth bubble collapses are presented in Fig. 2d and e, respectively, for two different events. The frames show how the bubble shrinks towards the surface and emits strong shock waves at collapse, with a clearly visible head wave indicating strong coupling of shock waves into the solid structure below the bubble. Shock wave emission was simultaneously measured using a needle hydrophone (Mueller-Platte) positioned about 3–4  mm from the bubble center.

The erosion patterns presented in the manuscript are a result of 1000 event repetitions, each repetition consisting of 100 ms of active ultrasound driving with no observable self-triggered acoustic cavitation and followed by the laser-induced bubble seeding at the end of this period. The final surface measurements were performed using a laser-scanning optical microscope in topographical imaging mode (confocal laser scanning), using Olympus LEXT™ OLS5100 microscope with an 20x magnification objective.

## 3 Results

**Fig. 3:**
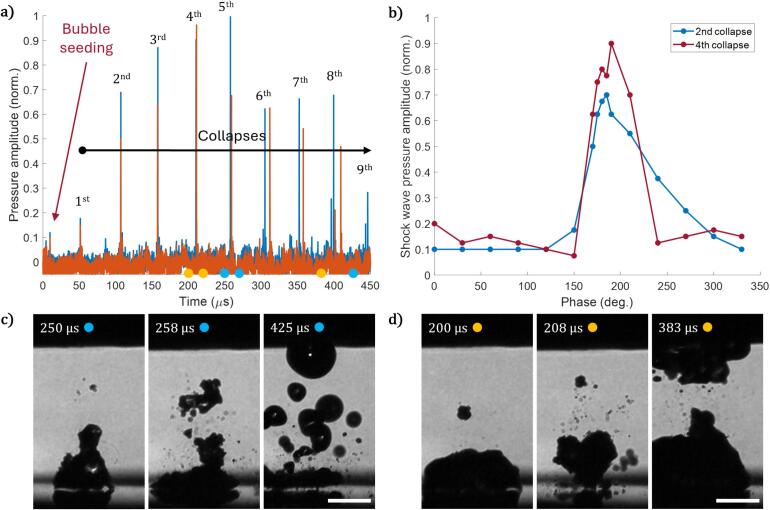
a) Acoustic emission from bubble nucleation to up to the 9th bubble (cloud) collapse, showing two examples of pressure traces at the same experimental parameters. Chosen frames from high-speed videos, corresponding to times marked with colored dots for the two events, are shown in panels c) and d). b) Shock wave pressure amplitude of the 2nd and 4th collapse as a function of the bubble generation phase φ. Corresponding videos are available in the Supplementary Material.

The lifetime of an optically seeded bubble without acoustic driving is about 1/5 of the sonotrode oscillation period. Therefore, we must first address the research question of phase delay between the sonotrode and the time delay Δt of the optically induced bubble seeding by varying the latter with respect to the acoustic driving. The time delay Δt is further referred to as the generation phase φ for clarity of interpretation and is calculated as $\varphi =360{}^{\circ}\Delta t/t_{0}$. Here, $t_{0}$ is the acoustic period duration. The generation phase is zero when bubble generation coincides with sonotrode passing through the rest position while moving downwards. For a phase of φ=180° and a similar experiment as shown in Fig. 2b (gap height of 0.50 mm) we obtain the pressure traces plotted in Fig. 3a (two repetitions performed under the same experimental parameters. The bubble generation and the successive bubble collapses generate an acoustic transient that increases in amplitude for the first 4–5 cycles. High-speed recordings confirm that until the 4th collapse the bubble remains a single entity and only breaks up afterwards. Chosen frames from high-speed videos, corresponding to times marked with colored dots for the two events, are shown in Fig. 3c and d. Peak pressure drops after bubble breaking, and multiple bubbles lead to multiple pressure peaks per collapse.

The pressure peaks are separated in time by about 50 µs, which is the period of acoustic driving, and the emission occurs in sync with the bubble or bubble cloud collapse. With the formation of scattered bubbles or a small bubble cloud, the sharp peaks split due to multiple shock wave emitters in the system, e.g., most peaks after the 300 µs time stamp. Given the established correlation between solid material erosion and near-surface bubble collapses [25], [31], [32], [33] and the collapse position variations explained in Section 2.2, Fig. 2, we have chosen to examine the pressures emitted from the 2nd and 4th collapses with respect to the generation phase in detail (Fig. 3b). Within a relatively narrow range of bubble generation phase, 170°<φ<210°, the pressures from both collapses peak. The pressure emitted at the fourth collapse peaks sharply, and the range of the phase φ=190°±10°. Interestingly, this phase is also favorable for acoustic bubble persistence as a single entity. Thus, the collapse of a single bubble emits stronger pressure waves than a cloud of similar volume. We expect that this phase regime is also the most erosive acoustic bubble regime.

**Fig. 4:**
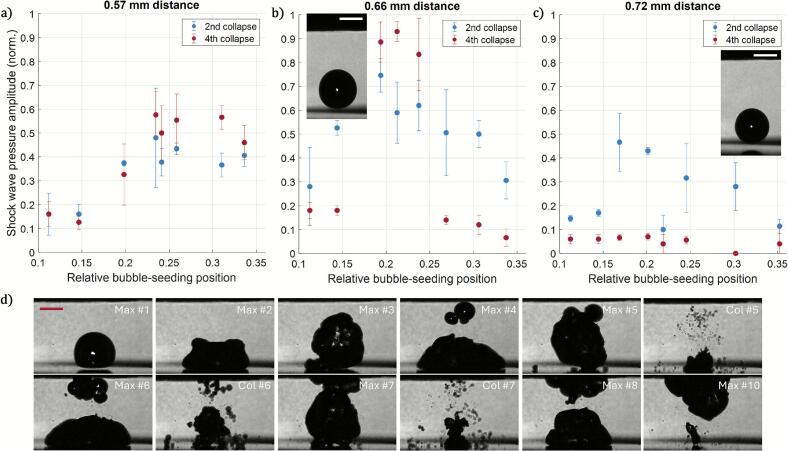
Collapse pressure amplitude at different relative bubble-seeding positions d_rel_ for three gap heights H (0.57 mm, 0.66 mm, and 0.72  mm), presented in panels a) to c), respectively. A maximum pressure is found for these three positions within 0.2 < d_rel_ < 0.25. The frame insets in b) and c) depict the bubble at the maximum volume reached within its first oscillation to compare the bubble size with the gap height, specifically at relative bubble-seeding positions of b) 0.306, and c) 0.245. d)Cavitation evolution over 10 acoustic cycles for H = 0.57 mm and relative bubble-seeding position of 0.235. After the fifth maximum (Max), collapses (Col) become progressively more scattered, and the bubble (cloud) tends to attach to the sonotrode surface. The scale bars are 200 µm. Corresponding videos are available in the Supplementary Material.

Next, we explore the position of the bubble seeding and the gap height on emitted pressures. A non-trivial pressure amplitude distributions forms within the gap between the sonotrode tip and the solid plate [34], [35]. Here, we limit the parameters where the bubble remains as a single entity persists and emits the strongest collapse shock waves. During experiments we have observed that a bubble cloud quickly covers the sonotrode tip if the laser-induced bubble dissipates to multiple smaller bubbles and attaches to the sonotrode. Therefore, we limited the gap sizes to be larger than the maximum bubble size during its first oscillation, thus that the bubble should stay close to the bottom half of the gap. Additionally, the laser focus was chosen in the bottom quarter of the gap, to promote bubble oscillations near the solid surface. The measurement points presented in Fig. 4 were each measured 2–10 times at the same experimental parameters to test and ensure the repeatability and reliability of the results. This is reflected by their respective error bars.

For the smallest gap height (Fig. 4a), we observe early formation of a bubble cloud already for d_rel_ > 0.3. Significantly smaller gaps than *H* = 0.57 mm result in bubbles that contact the sonotrode tip surface, leading to non-repeatable oscillations and bubble cloud formation. For the mid-sized gap height *H* = 0.66 mm, a clear maximum pressure amplitude is observed for the 2nd and 4th collapse, see Fig. 4b. Similarly, the second collapse leads to a single maximum for the largest gap height *H* = 0.72 mm as shown in Fig. 4c. Yet for the latter case, after the 2nd or 3rd oscillation the bubble disintegrates and the emitted pressure is either low or even below noise level of the hydrophone used, which is indicated then as a zero-pressure amplitude in the graph. Except for the largest gap distance, acoustic bubbles are highly likely to remain a single entity for at least 4 consecutive collapses for presented data in Fig. 4. An example of the cavitation evolution over 10 acoustic cycles is shown in Fig. 4d, which corresponds to gap height H = 0.57 mm and relative bubble-seeding position of 0.235 (data point presented in Fig. 4a). Single-entity behavior persists for 5 acoustic cycles, presented though chosen frames of bubble state close to its maximal (Max) or minimal volume (Col). We want to note that due to inherent variations in the experimental realization, some events do not exhibit an exact dynamic discussed and presented in Fig. 4d, e.g., due to initial asymmetries or a temporary presence of impurity within the gap. Said differences result in a varied number of bubble oscillations as a single-entity and varied pressure traces (as shown in Fig. 3a). Overall, we have established that the strongest bubble collapses should occur at the relative bubble-seeding position 0.2 < *d_rel_* < 0.25.

**Fig. 5:**
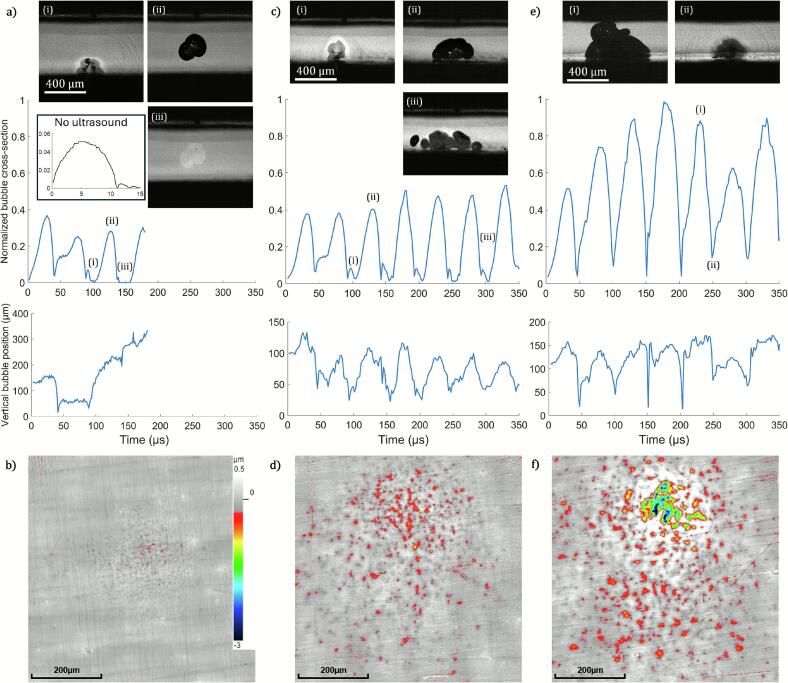
Overview of different bubble oscillation regimes as a function of the gap height. Each height leads to a distinct time-dependent bubble cross-section and normalized by the largest bubble (cross-section of 1.8·10^5^ µm^2^). Signature moments are shown in snapshots, and the final state of the aluminum surface is shown from surface topography measurements with a common depth scale. a) Gap height of H = 0.55 mm, the bubble stays near the plate surface for two acoustic cycles. The second collapse is closest to the plate surface (panel i). Later the bubble lifts towards the sonotrode surface (panel ii) and exhibits long-period minimal volume, nearly zero (panel iii). Inset-graph shows laser-induced bubble evolution without ultrasound driving for comparison, using the same vertical and horizontal scales. b) A smaller gap with H = 0.40 mm results in a single bubble that oscillates for longer time as a single bubble (panel ii) and reveals stronger and more collapses near substrate (panel i) but forms a cloud later (panel iii). c) The smallest gap with H = 0.35 mm, the bubble exhibits stable oscillations for the longest duration (panel i), and the strongest collapses, coupling surface waved into the substrate (panel ii, head-wave visible to the right). Full videos are available in the Supplementary Material.

Next, we explore the resulting cavitation erosion. For this we utilize a polished aluminum substrate as the lower solid surface. This material was chosen due to its’ malleability, a wide spectrum of industrial and scientific usage examples, and being used in in previous studies on cavitation erosion, e.g. Refs. [25], [32]. Three different bubble propagation regimes in the gap are presented in Fig. 5, featuring bubble cross-section area and bubble centroid position graphs as a function of time, selected high-speed camera frames, and laser-scanning confocal microscopy scans of the surface topography after 1000 consecutive experiments with unchanged experimental parameters. Each graph presents one experimental run and ends once the bubble splits and forms a cloud. Additionally, the graph shows the maximal bubble size and the kind of oscillation.

The first case, presented in Fig. 5a, corresponds to the largest gap height *H* = 0.55 mm, where the bubble first collapses towards the bottom solid surface, undergoes one oscillation near the surface (collapse in panel i), then leaves the surface (panel ii) and even remains in size below the resolution of the camera (panel iii) for a part of the oscillation cycle. During these periods the bubble does not dissolve but is a cavitation nucleus that is later in the acoustic cycle re-activated. After 1000 consecutive events there, the polished aluminum substrate has changed and is covered with small and isolated dimples. The dimples spread within a circle of approximately 200 µm diameter (Fig. 5b), each reaching a depth of around 0.5 µm.

The second case presented in Fig. 5c corresponds to the smaller gap height *H =* 0.40 mm. Here, the bubble first collapses towards the lower solid surface, then undergoes multiple oscillations, see second collapse in panel i. It retains the shape of a connected volume for multiple oscillations (panel ii) before it exhibits a strong fourth collapse occurring very close to the substrate (panel iii). The bubble cross-section of the second expansion is about 30  % larger than in the previous case. From the second collapse onwards, the bubble exhibits a frequency doubling behavior. Each strong collapse is followed by a small rebound, followed again by a larger expansion. After 1000 consecutive events, there is a marked change on the polished aluminum surface in the form of medium-sized dimples spread within an approximately 600 µm diameter (Fig. 5d). Dimples reach up to 1.0 µm in depths, creating a significantly increased deformation on the surface as compared to the first case.

The third case, presented in Fig. 5e, shows the bubble dynamics for the smallest gap height *H* = 0.35 mm. Here, the bubble first collapses towards the bottom solid surface, then undergoes multiple oscillations near the surface while it retains the connected volume form for multiple oscillations (panel i) and exhibits as the strongest collapse the fourth one that occurs in close contact with the substrate (panel ii). Here head waves are visible that indicate coupling of the shock waves from the liquid into the substrate. The bubble cross-sections are now considerably larger than in the previous case. Here the bubble reaches a 3-times larger size. Yet we do not observe a frequency doubling oscillation of the bubble. Instead, the strong collapse is followed immediately by a large rebound and the expansion phase lasts about twice as long as the collapse phase. It is also faster than the Rayleigh collapse time, which would be 25–30 µs for a spherical bubble of similar diameter. After 1000 repetitions of the experiment many pits in the shape of smaller dimples and large craters are found on the aluminum substrate (Fig. 5f). The damage occurs within a circular region with a diameter of approximately 600 µm. We find pits of up to 3.0 µm in depth, with the most significant damage concentrated within less-than 200 µm in diameter and centered around the laser focus. The measured high-activity erosion region caused by the acoustically driven laser-induced cavitation bubble is found to be much smaller that the sonotrode tip area, in a ratio of 1:100.

While we cannot rule out that the later collapses of the bubble cluster could contribute to the erosion, the narrow distance distribution of pits close to the laser seeding position suggests that the first few bubble oscillations contribute to the damage. If the later developing cloud that covers a much larger area with a diameter of up to 2 mm would contribute, then we would expect to see damage further away. Additionally, we observe that bubble oscillation regimes correspond to Fig. 4 cases, though each of the Fig. 5 cases occur at smaller gap heights. This is likely due to a different plate material and shape used for bubble dynamics experiments (glass plate) and erosion experiments (aluminium plate), resulting in different acoustic pressure distributions in the gap.

The Fig. 5d and f cases show asymmetry of the erosion patterns that can only be connected to the asymmetry of collapses, as the initial near-spherical bubble is generated with good repeatability and positional stability. Stronger and denser erosion patterns are found in the upper-central part of the surface scans. The area outside of the presented surface scans is nearly free of pits for these two gap heights. An asymmetric collapse was also found to be important in related studies in absence of a sound field [25], [33].

## 4 Conclusion

In summary, this work demonstrates that an individual acoustically driven cavitation bubble, optically seeded at a precise position and phase with respect to the sound field below a sonotrode tip, can reproducibly erode a solid substrate. By creating a laser-induced bubble at a phase of φ=190°±10° phase in a 20 kHz acoustic field and positioning the bubble at 20–25 % of the gap height closer to the substrate, sustained single‑entity bubble oscillations through four or more acoustic cycles were achieved. In this scenario, the fourth collapse delivers the strongest shock waves during the collapse. Systematic variation of the sonotrode–plate gap distance revealed three distinct erosion regimes: weak damage at large gaps, moderate dimple formations at intermediate gap heights and pronounced crater formation of up to 3.0 µm in depth for the smallest gaps (around *H* = 0.40 mm or smaller). High‑speed imaging combined with hydrophone measurements enabled a direct correlation of the collapse asymmetry and shock‑wave amplitudes with the observed surface topographies. This methodology opens a new avenue for controlled cavitation‑erosion studies in acoustic cavitation, offering spatial and temporal control over bubble dynamics and their erosive effects in a reproducible manner.

## CRediT authorship contribution statement

**Jaka Mur:** Writing – original draft, Resources, Methodology, Investigation, Conceptualization. **Vid Agrež:** Writing – original draft, Resources, Methodology, Investigation. **Claus-Dieter Ohl:** Writing – original draft, Resources, Methodology, Investigation, Conceptualization. **Rok Petkovšek:** Writing – original draft, Resources, Project administration, Investigation, Conceptualization.

## Declaration of competing interest

The authors declare that they have no known competing financial interests or personal relationships that could have appeared to influence the work reported in this paper.
